# A study protocol to evaluate the impact of a personal and domestic hygiene intervention on lead exposure in a community next to a mine dump

**DOI:** 10.1186/s12889-022-13439-8

**Published:** 2022-06-04

**Authors:** Charlotte Mokoatle, Angela Mathee, Renee Street, Vusumuzi Nkosi

**Affiliations:** 1 Department of Environmental Health, Faculty of Health Sciences, University of Johannesburg, Johannesburg, 2094 South Africa; 2 Environment and Health Research Unit, South African Medical Research Council, Johannesburg, 2094 South Africa; 3 School of Public Health, Faculty of Health Sciences, University of the Witwatersrand, Johannesburg, 2094 South Africa; 4 School of Health Systems and Public Health, Faculty of Health Sciences, University of Pretoria, Pretoria, 0001 South Africa

**Keywords:** Lead exposure, Mine dump, Intervention, Lead, Hygiene, Children, South Africa

## Abstract

**Background:**

Lead has been associated with adverse health effects, especially neurocognitive and behavioural effects, in children. Communities living close to mining land are at risk of elevated exposure to lead.

**Methods:**

This paper outlines a before and after intervention study protocol to evaluate the impact of a personal and domestic hygiene intervention on lead exposure in a community located adjacent to a mine dump. In each participating household, parents or guardians will be interviewed using a structured questionnaire to obtain information on socio-demographic characteristics, living conditions, domestic hygiene practices and potential alternative sources of exposure to lead. A registered nurse will collect hand wipe samples from children aged one to five years, for whom parental consent and where possible child assent has been obtained. Environmental dust samples will be collected from the floors and/or windowsills of children’s dwellings for lead content analysis. Soil samples will be collected from yards to determine lead content. An educational intervention will then be applied to the intervention group, including the engagement of households or guardians in an educational discussion on the sources, pathways of exposure, health effects of lead exposure and protective measures, with the aid of a specially designed educational brochure. Data will be analysed for descriptive and inferential statistics using Stata version 16.

**Discussion:**

The study will determine whether the intervention led to a reduction in indoor dust lead levels, and if shown to be effective, will inform the development of an awareness campaign to reduce lead exposure in communities located in close proximity to mine dumps.

**Trial registration:**

The study is retrospectively registered on ClinicalTrials.gov Protocol Registration and Results System with registration number NCT05265572 and first release date of 18^th^ February 2022.

## Background

Lead is a pervasive environmental toxicant and is associated with a spectrum of adverse health effects [1]. No safe threshold for lead exposure has been identified; however, the Centers for Disease Control and Prevention adopted a reference level of 5.0 μg/dL in 2016 for public health intervention concerning exposure to lead [2]. This level is related to the distribution of population and will be regularly reviewed. Notably, the World Health Organization has listed lead among the top ten toxic metals of public health concern [3]. Exposure to lead has been associated with behavioural disorders, especially in children who are particularly susceptible to its detrimental effects. In some parts of South Africa, residential areas have been developed very close to mine dumps [4] with an estimation of 1.6 million people living in settlements directly next to mine dumps [5, 6]. Mine dumps consist of a complex mixture of dust and particles, to which metals and other harmful substances may be attached. The dumps are major generators of wind-blown dust, which may contain metals such as lead, with potential risk to human health in exposed communities [4, 7, 8], posing a greater risk of elevated blood lead levels in children [9]. Children, especially those under the age of five years, are recognized to be a group that is particularly vulnerable to lead, both in terms of exposure and the health consequences [10]. Such detrimental health effects may include stomach cramps, kidney dysfunction, anaemia, constipation and high blood pressure [10]. Lead may also influence children's physical growth and body composition through several biological mechanisms [11].

## Methods

### Study design and population

**Fig. 1:**
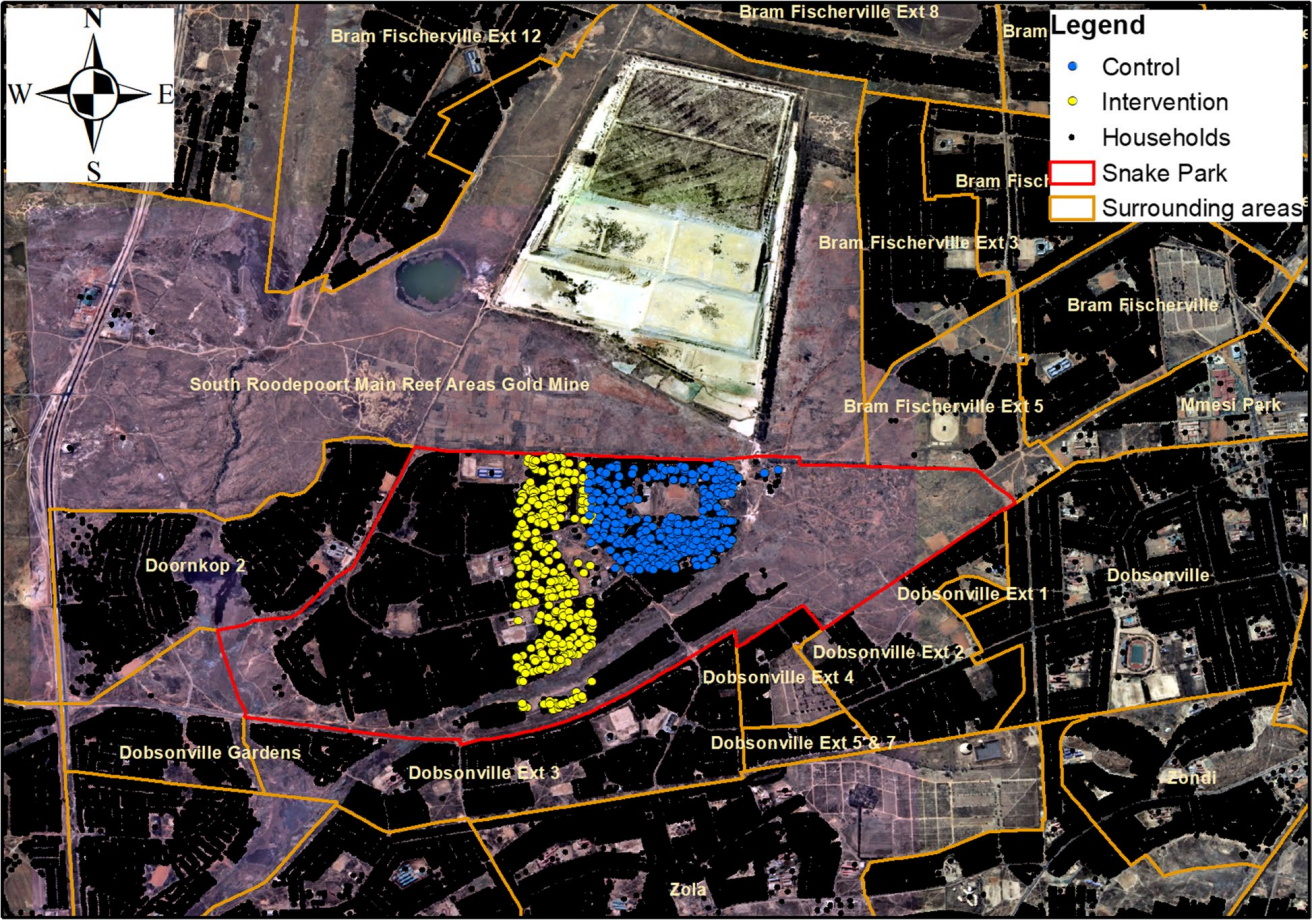
Map of Snake Park showing control and intervention group (source: Vusumuzi Nkosi, South African Medical Research Council)

A before and after intervention study will be conducted with a control and intervention group. This study follows a descriptive, correlational and experimental design [12]. The study area has a population of 4008 with 1124 households situated in Johannesburg Metropolitan Municipality adjacent to the Durban Roodepoort Deep Mine Dump. Figure 1 shows the intervention and control groups within the study area. The study targets children between the ages of one and five years. Children whose parents have not consented to any part of the research, or children who have not agreed, will be excluded from the study. Only parents/guardians over the age of 18 years will be included in the study.

The map of the study area reflecting household street names and stand numbers will be used to stratify single dwellings in close proximity (within 500 m) to the mine dump. Households located in the intervention group will be randomly selected and matched by structure and type (e.g. government-funded houses or non-government funded houses) with a dwelling in the control group.

### Determination of the sample size

Strata will be defined according to two main divisions within the catchment area. Systematic random samples will be taken within each division where every Xth dwelling will be sampled. Sample sizes were calculated at 80% power. The number of households per group is increased by 5% to account for possible loss to follow-up. To detect a mean difference in change from baseline to follow-up of -3 μg/dL between the control and experimental groups i.e., a lower lead level by 3 μg/dL in the intervention group compared to the control group, with 80% power and common standard deviation of 11 μg/dL, a sample of 213 households per group is required. To account for LTFU the number of households per group is adjusted to 224. Therefore 448 households are to be recruited and randomized to control and experimental groups accordingly.

### Data collection procedure

Data will be collected using multiple methods, namely, 1) a structured questionnaire; 2) hand dust wipe samples from children aged between one and five years; 3) floor and/or windowsill dust wipe samples; and 4) yard soil samples. These methods will be applied in two phases. In phase one, participants will be allocated into either the control or intervention group (see Fig. 1). Both the control and intervention groups will be interviewed, household floor and/or windowsill dust wipes will be taken, yard soil samples collected, and hand dust wipe samples taken from children in participating households. The intervention group will participate in the domestic hygiene demonstration and educational discussion. The intervention group will be allowed a period of three months to apply the domestic hygiene intervention. Each household will be given a diary to record their daily cleaning patterns. During the three months, a monthly reinforcement visit will be undertaken to follow-up and encourage the sustained application of the intervention. In phase two, after three months, both the intervention and control group will be visited to collect a repeat floor and/or windowsill dust sample, yard soil sample and hand dust wipe sample from children. Feedback will be obtained from the intervention group on the experience of the intervention using a checklist.

### Questionnaire-based interview (method 1)

A hard copy structured questionnaire will be administered through an interview with a parent or guardian to obtain information on living conditions, socio-economic status, domestic and personal hygiene practices and potential alternative sources of exposure to toxic metals. The questionnaire has been widely used in South Africa for blood lead surveys conducted by the Environment and Health Research Unit of the SAMRC.

### Hand dust wipe sample (method 2)

Hand dust wipes samples will be collected from assenting children aged between one and five years and/or whose parents/guardians have given consent to participate in the study. Ghost wipes will be used to wipe the hand from the heel of the hand above the wrist up to the fingertips. The left and right will be wiped following the same pattern on both the back and palm of the hand.

### Floor and/or windowsill dust wipe sample (method 3)

Samples of floor and/or windowsills dust will be collected during the baseline and post-intervention visits. The collection methods for floor and windowsill dust will be based on US Department of Housing and Urban Development guidelines for the evaluation and control of lead-based paint hazards in housing [13]. Floor and/or windowsill dust samples will be collected from the room where children spend most of their time. If the room contains a carpet, then floor dust samples will be collected from the kitchen. If there are no windowsills in the house selected, then the floor dust samples will be collected. An area to be sampled will be a rectangle or square measuring one square meter that will be demarcated using a commercial template. Disposable dust wipes will be used to collect dust samples.

### Soil sample collection (method 4)

Soil samples will be collected in the yard of the selected households in both the intervention and control groups (up to a depth of 10 cm) in accordance with the US EPA methods. Surface soil samples will be stored in a transparent plastic bag. To reduce moisture in the collected soil samples, the samples will be dried for 24 h at 40 °C. The samples will be crushed and sieved to a particle size of less than 250 µm.

### The intervention

#### Personal hygiene intervention

**Fig. 2:**
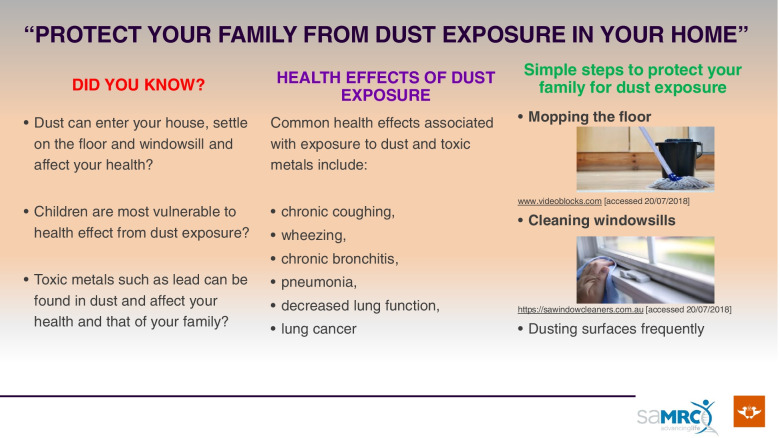
Educational pamphlet

At the end of the questionnaire-based interview, blood collection and environmental sample collection, participants (parents/guardians) will be engaged in an educational discussion for approximately 30 min, with the aid of a specially designed educational pamphlet (Fig. 2). The pamphlet contains information about health effects potentially associated with dust exposure from mine dumps and the personal hygiene and domestic hygiene steps that may be followed to reduce dust exposure. The pamphlet will be pre-tested during the pilot study and refined as necessary.

#### Domestic hygiene intervention

The researcher, through a demonstration to participants (parents/guardians), will conduct a domestic hygiene intervention. During the demonstration, participants will be guided on optimal cleaning of windowsills, floors and other surfaces with water and detergent, and advised on an appropriate method of cleaning. The Centers for Disease Control and Prevention recommends a continuing program of wet mopping and damp dusting of all hard surface floors, stairs, windowsills and baseboards at least twice a week, to keep lead-contaminated house dust down to a minimum. Participants will be provided with a package of cleaning materials (mop, bucket, detergent, hand wash liquid soap and cloth for wet dusting) which will be used during the demonstration and retained by participants to facilitate the sustainability of the intervention during and after the study. The cleaning materials will be replenished on follow-up visits.

### Data management and analysis

#### Hand dust wipe sample analyses

The samples will be stored in a sealable plastic bag and transported by the research team to a designated laboratory at the Doornfontein Campus, University of Johannesburg for analyses of Lead content.

#### Floor and/or windowsill sample analyses

Each floor and/or windowsill dust sample will be sent by the researcher to a designated laboratory at the Doornfontein Campus, University of Johannesburg for analyses of lead content. Lead dust levels will be determined using inductively coupled plasma mass spectrometry after appropriate sample preparation. The United States Environmental Protection Agency’s dust lead standards, 10 µg/ft^2^ for floors and 100 µg/ft^2^ for windowsills will be applied in this study.

#### Soil sample analyses

Each soil sample will be analysed for lead content for 60 s using an X-ray fluorescence spectrometer, available at the SAMRC Environment and Health unit designated lab. A trained technician at the SAMRC will do the analysis. The mean, median and range of the levels of lead will be recorded for the control and intervention groups. Mathee et al. found severe and extensive lead contamination of garden soil which warranted action to investigate further and protect the health of the affected communities [14].

### COVID-19 health and safety precautions

The study will adopt precautionary procedures to address and mitigate the spread of Covid-19 between field staff and community members/study participants. These procedures will be guided by the ‘COVID-19 OCCUPATIONAL HEALTH AND SAFETY MEASURES IN WORKPLACES COVID-19 (C19 OHS), 2020’, as promulgated by the South African Department of Employment and Labour. Amongst other aspects, if field staff are sick or have symptoms associated with COVID-19 prior to or during data collection, they will not be permitted to participate in data collection and will be instructed to self-quarantine. Field staff members will wear a face mask when reporting for duty and during interactions with study participants. In addition, requests will also be made for study participants to also wear a face mask. If the participant does not have a face mask, the field staff shall provide them with one. The procedure will also take into account the practice of social distancing during field staff interactions, as well as interactions between field staff and study participants by having individuals stand 1.5–2 m apart. Where study activities make social distancing not practicable, field staff members will put on a face shield, in addition to wearing a face mask. Additionally, a hand sanitiser with at least 70% alcohol content will always be present and made available for use by the field staff and study participants. More specifically, field staff members will be required to use the hand sanitiser before and after each interaction with one another and with study participants, as well as clean and disinfect surfaces and equipment that is touched during data collection.

### Data analysis plan

Descriptive statistics such as means, medians, standard deviations, interquartile and 95% confidence intervals will be used to describe lead levels and other continuous outcomes. Frequencies and proportions with 95% confidence intervals will be calculated for categorical variables. The paired t-test will be used to compare the difference in lead levels within groups between baseline and follow-up. The two-sample t-test will be used to compare the mean lead levels between groups. McNemar’s test will be used to compare the children’s health outcomes between baseline and follow-up. The change from baseline to follow-up will be modelled, with a group main effect, with adjustment for other covariates. A linear mixed-effects model will be used to model the change in lead levels resulting from the hand dust wipes, with the main effect for the group. Adjustments will be made for lead levels in homes, as well as other important baseline characteristics, including variables from the administered questionnaire. The model will be adjusted for clustering within the household. Depending on the level of missingness, imputation of follow-up data may be conducted, and analysis rerun as sensitivity analysis. Statistical significance is taken at a 5% level. All analyses will be conducted using STATA 16.

### Possible outcomes

It is expected that the study will provide baseline data on children’s exposure and general household exposure to mining dust lead. The study will also reveal the effectiveness (or not) of an educational and domestic hygiene intervention in reducing childhood lead levels in a settlement located close to a mine dump. Should the study reveal a significant, positive effect, it will demonstrate the potential for a simple domestic and environmental intervention to protect communities living close to mine dumps (and potentially other sources of pollution) from exposure to associated toxic substances, specifically lead.

### Results dissemination

We aim to disseminate the results from the study to the participants, and policymakers in the health sector through conference and stakeholder presentations and official report. Furthermore, publish the findings of the study in peer-reviewed publications.

### Study progress

The study has received scientific approval and ethical clearance to commence with the research project. The study pilot stage is completed, and data collection has commenced.

## Discussion

A research study has shown that there has been significant growth in the number of households and housing developments, close to the TSFs in South Africa [15]. Furthermore, the study stated that the implications for many residences downwind mine dumps justify ongoing monitoring efforts and educational programmes [14]. Occasionally, two strategies are implemented to reduce pollution from such mine dumps i.e., spraying of mine tailings dumps with water and rehabilitation through planting grass. However, these strategies are often deemed ineffective, since grass withers during the dry season in particular, and sprayed water is rapidly absorbed or evaporated. The current extent of environmental contamination from mine dumps and potential health impacts in nearby areas has been researched, the findings showed that participants identified mine dumps as the biggest source of dust [16]. Other studies have explored the acceptability and feasibility of changing housekeeping behaviour to reduce childhood lead exposure [17]. A study by Feit et al. found that certain personal and domestic hygiene interventions were feasible and acceptable to the study community and were significantly associated with a reduction in indoor dust lead concentrations [16]. However, there is a need to conduct further trials in developing countries to determine the effectiveness of interventions for the prevention of lead [18]. A review by Nussbaumer-Streit et al. found that educational interventions were not effective in reducing blood lead levels and dust control interventions may lead to little or no difference in blood lead levels [19]. However, the quality of evidence was moderate to low, meaning that future research is likely to change these results. No further research has yet been undertaken to evaluate the impact on childhood lead levels of a community-based lead exposure reduction programme focused on personal and domestic hygiene. In particular, there remains a gap in knowledge regarding household hygiene interventions to reduce exposure to lead in communities living close to mine dumps in South Africa, particularly among children. This study, which intends to evaluate the impact of a household hygiene intervention comprised of an educational and domestic household hygiene intervention to reduce mining-related dust exposure, will address this need. The study holds the potential for a cost-effective measure to reduce exposure to mining-associated toxic substances, for hundreds of thousands of households living in the shadow of mining operations and other sites of elevated exposure to toxic substances from industrial sources.

## Data Availability

Data sharing is not applicable to this article as no data sets have been generated or analysed during the current study.

## Abbreviations

TFS: Tailing Storage Facilities
SAMRC: South African Medical Research Council
U: University of Johannesburg
NRF: National Research Council
